# 4-1BB Signaling Promotes Alveolar Macrophages-Mediated Pro-Fibrotic Responses and Crystalline Silica-Induced Pulmonary Fibrosis in Mice

**DOI:** 10.3389/fimmu.2018.01848

**Published:** 2018-09-10

**Authors:** Yiping Lu, Chao Li, Sitong Du, Xi Chen, Xinning Zeng, Fangwei Liu, Ying Chen, Jie Chen

**Affiliations:** Division of Pneumoconiosis, School of Public Health, China Medical University, Shenyang, China

**Keywords:** 4-1BB, alveolar macrophages, crystalline silica, pro-fibrotic response, pulmonary fibrosis

## Abstract

Silicosis is caused by exposure to crystalline silica (CS). We have previously shown that blocking 4-1BB signaling attenuated CS-induced inflammation and pulmonary fibrosis. However, the cells that express 4-1BB, which plays a vital role in promoting fibrosis, are still unknown. In this study, we demonstrated that the expression of 4-1BB is elevated in alveolar macrophages (AMs) in the lungs of CS-injured mice. CS exposure also markedly enhanced the expression of 4-1BB in macrophage-like, MH-S cells. In these cells, activation of the 4-1BB signaling with an agonist antibody led to upregulated secretion of pro-fibrotic mediators. Consistently, blocking 4-1BB downstream signaling or genetic deletion of 4-1BB alleviated pro-fibrotic responses *in vitro*, while treatment with a 4-1BB fusion protein promoted pro-fibrotic responses. *In vivo* experiments showed that blocking 4-1BB signaling decreased the expressions of pro-fibrotic mediators and fibrosis. These data suggest that 4-1BB signaling plays an important role in promoting AMs-mediated pro-fibrotic responses and pulmonary fibrosis. Our findings may provide a potential molecular target to reduce CS-induced fibrotic responses in occupational lung disease.

## Introduction

Silicosis is a progressive, occupational lung disease, caused by long-term inhalation of crystalline silica (CS) (1). On account of the poor surveillance, silicosis remains a worldwide burden and is particularly prevalent in developing countries (2). Silicosis is characterized by chronic inflammation and progressive pulmonary fibrosis (3). Progression of the disease is accompanied with aggravated damage to lung function, even after workers have left dusty occupational environments, eventually resulting in disability and death (4). Many preventive measures have been set up to counter this disease, however, the incidence and prevalence of silicosis are still increasing (5). Therefore, effective therapeutics for silicosis are still needed.

Crystalline silica injures the lung tissues, leading to the recruitment, proliferation, and activation of immune and non-immune cells, including macrophages, CD4+ T cells, epithelial cells, and fibroblasts, which are involved in tissue repair (6). Since CS cannot be discharged from the lungs, inflammatory responses persist, leading to the subsequent dysregulation of the wound-healing response and the development of pathological pulmonary fibrosis (7). Alveolar macrophages (AMs) that can engulf CS are the first line of defense in immunity and exhibit a vital regulatory role at all stages of silicosis (8). They can secrete pro-inflammatory and pro-fibrotic cytokines, chemokines, and matrix metalloproteinases (MMPs) in response to CS (9). These mediators can create a pro-fibrotic environment that: (i) magnifies epithelial damage; (ii) increases the recruitment of immune cells, including monocytes, CD4+ T cells, and fibrocytes; and (iii) enhances the deposition of extracellular matrix (ECM) protein (7, 10, 11). Therefore, a reduction in AMs-mediated pro-fibrotic responses may be a plausible strategy for treating CS-induced pulmonary fibrosis.

The co-stimulatory molecule, 4-1BB [CD137, tumor necrosis factor receptor superfamily 9 (TNFRSF9)], belongs to the TNFRSF and can interact with 4-1BB ligand (4-1BBL) to play a role in innate and adaptive immune responses (12). Additionally, 4-1BB is expressed on a variety of hematopoietic cells including activated CD4+ T cells, natural killer cells, and macrophages, and is also inducible on non-hematopoietic cells, such as endothelial cells, epithelial cells, and microglia (13, 14). The crosslinking of 4-1BB can enhance the survival and proliferation of activated T cells, Th1 polarization, and cytokine production (15). An agonist-4-1BB mAb seems to aggravate the development of inflammatory metabolic diseases and infectious diseases (13, 16). As our previous study demonstrated, blockade of 4-1BB signaling may decrease CS-induced inflammation and pulmonary fibrosis *in vivo* (17). Nevertheless, the role (including any regulatory mechanisms) and the profile of expression of 4-1BB in CS-induced pulmonary fibrosis are still unknown.

Here, we show that 4-1BB is expressed on AMs in the lungs of CS-injured mice. Additionally, CS stimulation could induce 4-1BB expression on macrophage-like MH-S cells. Using these cells as a model of AMs, we show that 4-1BB signaling promoted the release of pro-inflammatory and pro-fibrotic cytokines, chemokines, and MMPs. Consistent with this, blockade of 4-1BB signaling alleviated pro-fibrotic responses *in vitro*. Further, pharmacological inhibition of 4-1BB signaling reduced pulmonary fibrosis responses *in vivo*. Our data identify an encouraging function of 4-1BB signaling in AMs-mediated pro-fibrotic responses and CS-induced pulmonary fibrosis.

## Materials and Methods

### Animals and Treatments

Animal studies were performed in accordance with the Animal Care and Use Committee at China Medical University and complied with the National Institute of Health (NIH) Guide for the Care and Use of Laboratory Animals. Six- to eight-week-old female C57BL/6 mice were obtained from SLAC Laboratory Animal Co. Ltd. (Shanghai, China), fed with standard laboratory chow diet and housed in colony cages under 12 h light/12 h dark cycles. CS particles (Min-U-Sil 5 ground silica; size distribution: 97% <5 μm diameter, 80% <3 μm diameter; median diameter 1.4 µm) were obtained from the U.S. Silica Company (Frederick, MD, USA) and prepared as previously described (17). CS-injured mice were intratracheally instilled with a 3 mg/50 μL CS suspension. Control mice received 50 µL sterile saline.

#### For the Expression of 4-1BB in Lung Cells

Mice were randomly divided into CS-injured and control groups (3–4 mice per group). The lungs were harvested and prepared for flow cytometric analyses after exposure of mice to CS for 7 days.

#### For 4-1BB Fusion Protein Treatment

100 or 50 µg of 4-1BB fusion protein (4-1BBIg; cat: 50811-M02H, Sino Biological Inc., Beijing, China) per mouse or 100 µg isotype IgG (human IgG1; Sino Biological Inc.) were injected intraperitoneally into mice, on days 1 and 4 after CS administration (3–4 mice per group). The specific methods used to treat mice have been described previously (17). Lung tissues were collected for western blot and enzyme-linked immunoassay (ELISA) analyses at 7 days after CS instillation.

#### For NQDI 1 Treatment

A dosage of 10 mg/kg bodyweight of NQDI 1 (Tocris Bioscience, Bristol, UK) or an equal volume of vehicle was intraperitoneally (i.p.) administered into mice after CS instillation (10 mice per group). Detailed methods have been outlined in the previous article (17). Lung tissues were harvested and used for immunohistochemical and western blot analyses after 7- and 56-days exposure to CS.

### Cell Culture

MH-S cells, a cell line of AMs, which have been widely used to mimic the function of AMs (18), were purchased from BeNa Culture Collection (Beijing, China). MH-S cells were cultured in RPMI 1640 medium supplemented with 10% heat-inactivated FBS, 100 U/mL penicillin, 100 µg/mL streptomycin, 10 mM HEPES, and 1 mM sodium pyruvate. Cells were incubated in a humidified atmosphere of 5% CO_2_ at 37°C.

### 4-1BB ShRNA Silencing

Lentivirus constructed with shRNA directed against mouse 4-1BB in pHBLV-U6-puro vectors, was prepared by Hanbio Biotechnology Co., Ltd. (Shanghai, China). The 4-1BB shRNA sequence is shown in Table 1. MH-S cells were transfected at a multiplicity of infection (MOI) = 10, which was indicated by immunofluorescence (Figure S3A in Supplementary Material). MH-S cells were transfected with lentiviral control vectors (Len-cont.) or vectors with shRNAs against 4-1BB (sh-4-1BB) in medium with 2 µg/mL polybrene for 24 h. After transfection, medium containing lentivirus was removed, and fresh medium was added. The cells were selected in the medium with 8 µg/mL puromycin after transfection for 48 h. The selected cells were stored frozen, passaged, and used for experiments.

**Table 1:** Sequences.

*Mus musculus*gene name	Top strand	Bottom strand
Sh-control	gatccgTTCTCCGAACGTGTCACGTAAttcaagaga TTACGTGACACGTTCGGAGAAttttttc	aattgaaaaaaTTCTCCGAACGTGTCACGTAAtctcttgaa TTACGTGACACGTTCGGAGAAcg
Sh-4-1BB	GatccGAAACCTGTAGCTTGGGAACATTTAATTCAA GAGATTAAATGTTCCCAAGCTACAGGTTTTTTTTTc	aattgAAAAAAAAACCTGTAGCTTGGGAACATTTAAT CTCTTGAATTAAATGTTCCCAAGCTACAGGTTTCg

	**Forward 5′-3′**	**Reverse 5′-3′**

*Tnfrsf9*(4-1BB)	CGTGCAGAACTCCTGTGATAAC	GTCCACCTATGCTGGAGAAGG
*Mmp9*	CTGGACAGCCAGACACTAAAG	CTCGCGGCAAGTCTTCAGAG
*Mmp12*	CTGCTCCCATGAATGACAGTG	AGTTGCTTCTAGCCCAAAGAAC
*Ccl2*	TTAAAAACCTGGATCGGAACCAA	GCATTAGCTTCAGATTTACGGGT
*Gapdh*	CAATGTGTCCGTCGTGGATCT	GTCCTCAGTGTAGCCCAAGATG

### Cell Experiments

#### The Expression of 4-1BB in MH-S Cells

MH-S cells were plated into 6-well plates (5 × 10^5^ cells per well) 24 h before the treatments. Complete medium with 10% FBS was then changed with new RPMI 1640 medium. MH-S cells were treated with CS (50 µg/cm^2^) or saline for 12 h. The harvested cells were used for western blot and real-time polymerase chain reaction (PCR) analyses.

#### Activation of 4-1BB Signaling

MH-S cells were prepared as previously described above. To examine the expression of IL-1β, MH-S cells were pretreated with lipopolysaccharide (LPS, 25 ng/mL, Sigma-Aldrich Chemical Company) for 3 h (Figure 4J). Agonist 4-1BB mAb (10 µg/mL; cat: MAB9371, R&D Systems, Minneapolis, MN, USA) (19) was used to detect the effect of 4-1BB signaling on macrophages 2 h before CS exposure. Isotype IgG (10 µg/mL, Sino Biological Inc.) was used as a control. After exposure to CS for 12 h, the culture supernatant was collected for ELISA analysis, and harvested cells were lysed for western blot and real-time PCR analyses.

#### Blockade of 4-1BB Signaling

Transfected (Len-cont. and sh-4-1BB) MH-S cells were seeded into 6-well plates (5 × 10^5^ cells per well) 24 h prior to the treatments. To detect the expression of IL-1β, transfected MH-S cells were pretreated with LPS for 3 h (Figure 5L). Cells then were cultured with or without NQDI 1, 4-1BBIg (cat: 50811-M02H), or IgG1 for 2 h and then incubated with CS (50 µg/cm^2^) for 12 h. NQDI 1, an inhibitor of apoptosis signal-regulating kinase (ASK)-1 that is downstream of the 4-1BB signaling, was added at a concentration of 10 µM and effects on 4-1BB signaling determined by western blot analysis (Figure S4 in Supplementary Material) (20). Additionally, 4-1BBIg, which interacts with 4-1BBL to suppress 4-1BB, was added at a dosage of 10 µg/mL (17, 21). IgG1 (10 µg/mL) was used as a control. Cells and culture medium were collected and used to detect changes in 4-1BB signaling and the level of pro-fibrotic cytokines.

### Flow Cytometry Analysis

Single-cell suspensions of lungs were prepared from mice as previously described (22). To reduce non-specific antibody binding, cells were incubated with a Fc receptor block (BD Pharmingen, San Jose, CA, USA). Then lung single-cell suspensions were stained with antibodies to CD45 (BD Pharmingen), F4/80 (Miltenyi Biotech, Bergisch Gladbach, Germany), CD11c (BD Pharmingen), CD137 (eBioscience™, San Diego, CA, USA), CD137L (eBioscience™), CD4 (BD Pharmingen), CD44 (BD Pharmingen), and CD62L (BD Pharmingen). For MH-S cells, the cell surface was stained by CD137 or CD137L antibodies after Fc blocking. Isotype-matched mAb were used as negative controls. A FACS Canto II flow cytometer (BD Biosciences, Franklin Lakes, NJ, USA) and FlowJo V10 software were used to analyze the expression of 4-1BB on lung cells.

### Western Blot Analysis

Western blotting was performed essentially as previously described (17). The expression of CD137 (1:500, rat, Abcam, Cambridge, UK), ASK1 (1:2,000, rabbit, Abcam), phospho-ASK1 [1:500, rabbit, Cell Signaling Technology (CST), Danvers, MA, USA], p38 (1:1,000, rabbit, CST), phospho-p38 (1:1,000, rabbit, CST), JNK (1:1,000, rabbit, CST), phospho-JNK (1:1,000, rabbit, CST), IκBα (1:1,000, rabbit, CST), phospho-IκBα (1:1,000, rabbit, CST), MMP9 (1:1,000, rabbit, Abcam), MMP12 (1:2,000, rabbit, Abcam), and β-actin (1:1,000, rabbit, CST) in MH-S cells or lung tissues was measured by western blot. Protein expression was normalized to β-actin.

### Real-Time PCR Analysis

Total RNA was extracted from cultured cells with TRIzol (Invitrogen) following the manufacturer’s instructions, and cDNA was synthesized using a Prime Script RT-PCR Kit (Takara, Kusatsu, Japan). Quantitative real-time PCR assays with a SYBR Premix ExTaq Kit (Takara) were performed by a 7500-sequence detector. The specific primer sequences used were shown in Table 1. GAPDH was used as the normalizing gene for determining ΔC_T_ values. Fold changes in gene expression were compared with 2^−ΔΔCT^ values.

### Cytokine Quantification

Culture supernatants were collected and centrifuged at 2,000 rpm for 10 min after 12 h culture. The supernatants were stored at −80°C and used for subsequent experiments. Lung lysates of different mice groups were diluted to a concentrate of 1 µg/µL with tris-buffered saline. The cytokines, interleukin (IL)-1β, IL-6, tumor necrosis factor (TNF)-α, and monocyte chemoattractant protein (MCP)-1, were measured by ELISA (R&D) according to the manufacturer’s instructions.

### Immunohistochemistry

After deparaffinization in xylene and subsequent rehydration in graded alcohol series, tissue sections were blocked by exposure to 3% H_2_O_2_ and boiled in a citrate buffer (pH 5.9–6.2) at 95°C for 20 min. After blocking in 5% BSA for 1 h at room temperature, tissue sections were incubated at 4°C overnight with antibodies to CD68 (1:200, rat, Abcam), MMP9 (1:200, rabbit, Servicebio, Wuhan, China), MMP12 (1:50, rabbit, Abcam), or collagen I (1:200, rabbit, Abcam). The sections were washed with phosphate-buffered saline, and then incubated with horseradish peroxidase secondary antibodies (Santa Cruz Biotechnology, Dallas, TX, USA) at room temperature for 2 h. A DAB kit (Santa Cruz) was used to perform the positive staining. Nuclei were stained by hematoxylin.

### Statistical Analysis

Two data sets were compared by Student’s *t*-test. Differences among groups were assessed using one way-ANOVA followed by a Student–Newman–Keuls test. Results were expressed as mean ± SEM. Significance was defined as *p* ≤ 0.05.

## Results

### 4-1BB Is Increased in AMs from CS-Injured Mice

**Figure 1:**
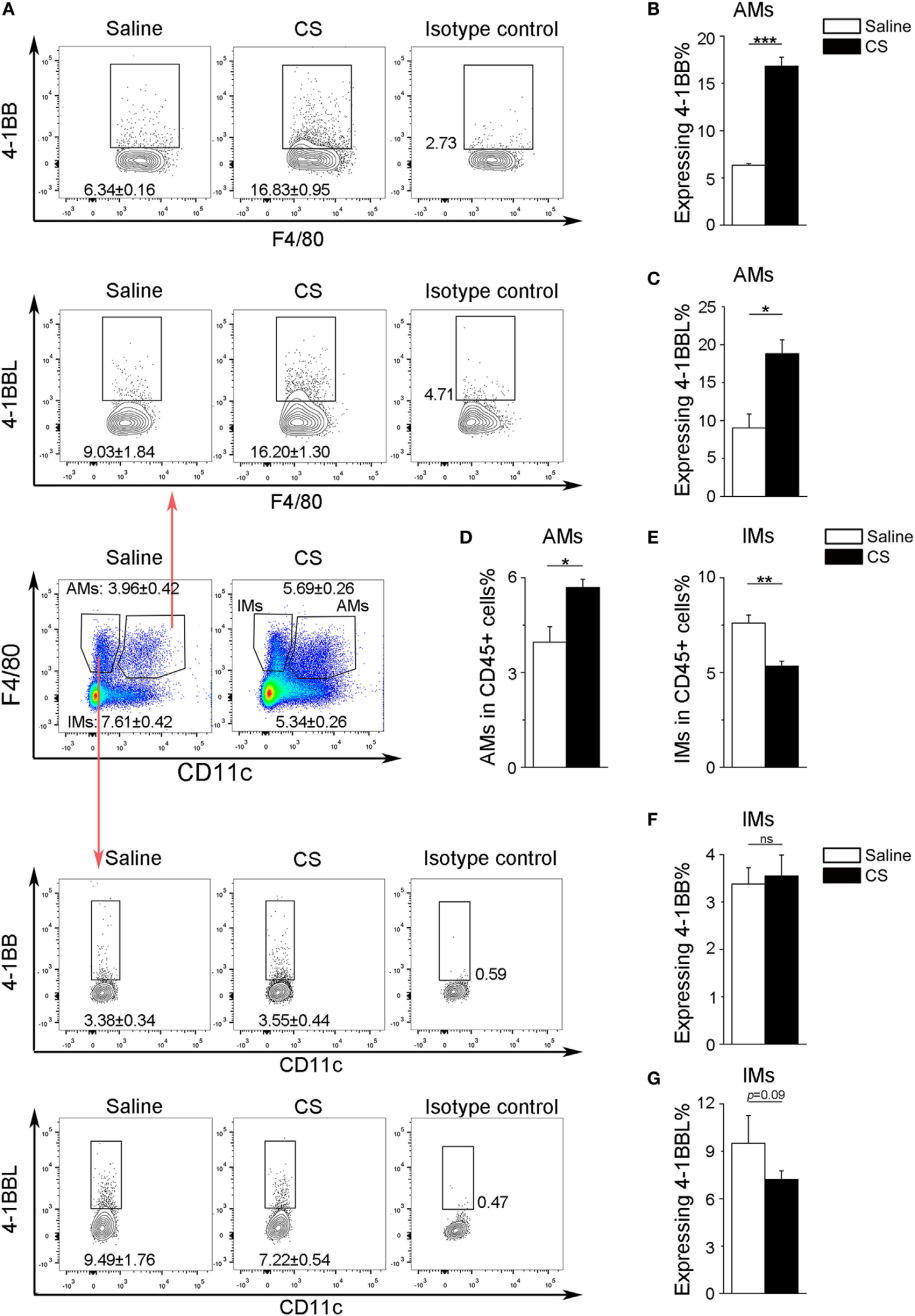
Expression of 4-1BB (CD137) and 4-1BBL (CD137L) on pulmonary macrophages. After 7-days exposure to crystalline silica, mice were sacrificed. The lungs were prepared as single-cell suspensions for flow cytometric analyses (*n* = 3–4). **(A)** Representative plots of flow cytometric analyses for 4-1BB and 4-1BBL on alveolar macrophages (AMs) and interstitial macrophages (IMs). **(B,C)** The percentage of AMs expressing 4-1BB and 4-1BBL. **(D,E)** The frequency of AMs and IMs in CD45+ cells from the lungs. **(F,G)** The percentage of IMs expressing 4-1BB and 4-1BBL. The experiments were performed twice with similar results. Data were shown as mean ± SEM (*p ≤ 0.05; **p ≤ 0.01; ***p ≤ 0.001; ns, not significant).

**Figure 2:**
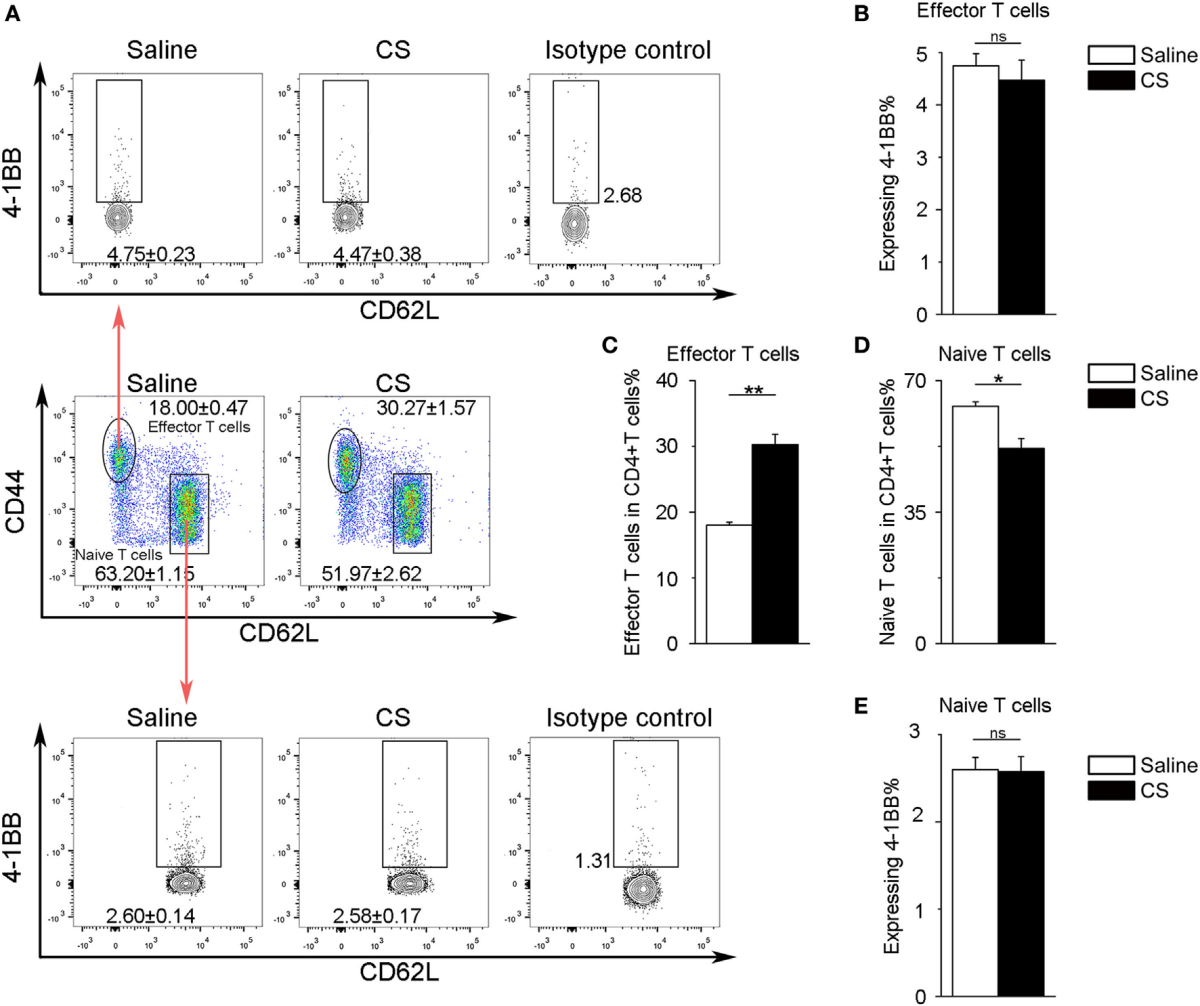
Expression of 4-1BB on CD4+ T cells. **(A)** Representative plots of flow cytometric analyses for 4-1BB on effector and naïve T cells. **(B)** The percentage of effector T cells expressing 4-1BB. **(C,D)** The frequency of effector and naïve T cells in CD4+ T cells from the lungs. **(E)** The percentage of naïve T cells expressing 4-1BB. The experiments were performed twice with similar results. Data were shown as mean ± SEM (*p ≤ 0.05; **p ≤ 0.01; ns, not significant).

In our previous study, we found that the expression of 4-1BB was significantly elevated in the lungs of CS-injured mice compared to controls after CS instillation (17). However, the types of cells expressing 4-1BB, which plays a vital role in promoting fibrosis, are still unknown. To examine the cellular expression of 4-1BB, we collected lungs to make single-cell suspensions 7 days after mice were exposed to CS. The percentage of AMs (F4/80+CD11c+) in CD45+ cells increased in CS-injured mice, while lung interstitial macrophages (IMs, F4/80+CD11c−) decreased (Figure 1A middle panels, Figures 1D,E). AMs showed a markedly higher expression of 4-1BB in CS-injured mice than controls. However, a difference between CS-injured and control mice in the expression of 4-1BB by IMs was not noted (Figures 1A,B,F). Macrophages, as antigen-presenting cells, express 4-1BBL, which activates T cells (23). Thus, we detected the expression of 4-1BBL on AMs and IMs in mice, after exposure to CS. The percentage of AMs expressing 4-1BBL was remarkably enhanced in CS-injured mice compared with controls, with no significant variation in 4-1BBL expression on IMs (Figures 1A,C,G). We also tested the expression of 4-1BB on CD4+ T cells. The frequency of effector T cells (CD44+CD62L−) among CD4+ cells increased in mice after exposure to CS, whereas naïve T cells (CD44−CD62L+) declined (Figure 2A middle panel, Figures 2C,D). The percentage of effector and naïve T cells expressing 4-1BB in CS-injured mice did not increase significantly (Figures 2A,B,E). These data indicate that AMs may be predominantly responsible for the increase of 4-1BB expression in the lung of CS-injured mice.

### MH-S Cells Express 4-1BB After Exposure to CS

**Figure 3:**
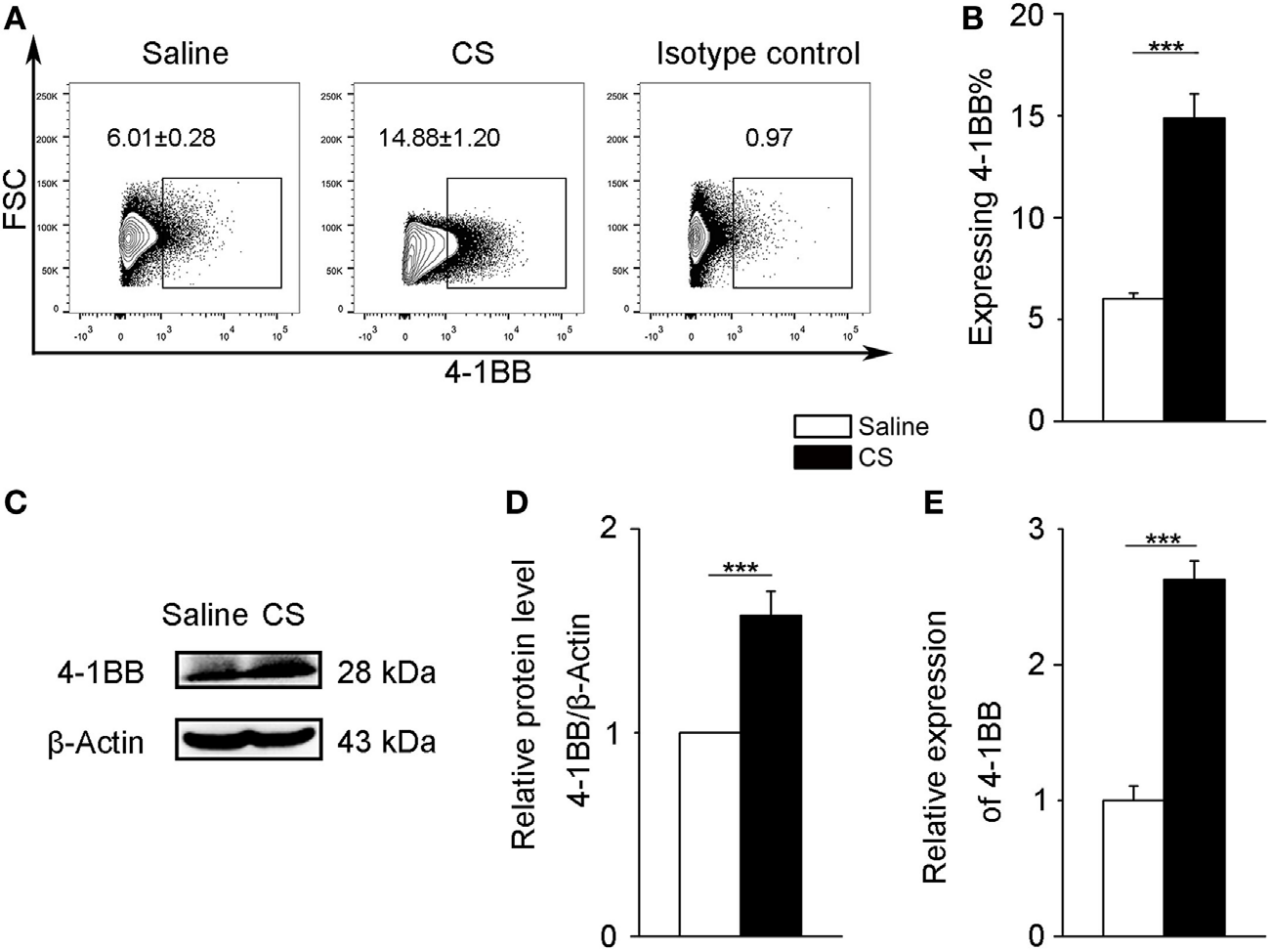
4-1BB expression on the mouse alveolar macrophage cell line, MH-S. MH-S cells were treated with crystalline silica (50 µg/cm^2^) or saline for 12 h. **(A,B)** The percentage of MH-S cells expressing 4-1BB (*n* = 4). **(C)** Western blot analysis of 4-1BB protein from whole cell lysates. **(D)** Quantification of the 4-1BB protein level relative to that of β-actin is shown (*n* = 3). **(E)** Total RNA was isolated to analyze 4-1BB mRNA expression (*n* = 4) relative to GAPDH. The data were representative of three independent experiments. Data were expressed as mean ± SEM (***p ≤ 0.001).

To further verify 4-1BB expression on macrophages after CS stimulation, we chose a mouse alveolar macrophage cell line, MH-S cells, which has been previously used in CS experiments (24). We treated MH-S cells with CS or saline for 12 h. The expression of 4-1BB and 4-1BBL was upregulated in MH-S cells exposed to CS (Figures 3A,B; Figures S2B,C in Supplementary Material). The protein level of 4-1BB significantly increased after exposure to CS (Figures 3C,D). We then used real-time PCR analysis to detect the 4-1BB mRNA expression. The relative expression of 4-1BB mRNA in MH-S cells cultured with CS was dramatically elevated (Figure 3E). The results suggest that CS could induce macrophages to express 4-1BB.

### 4-1BB Agonists Exaggerate the Pro-Fibrotic Responses of Macrophages

**Figure 4:**
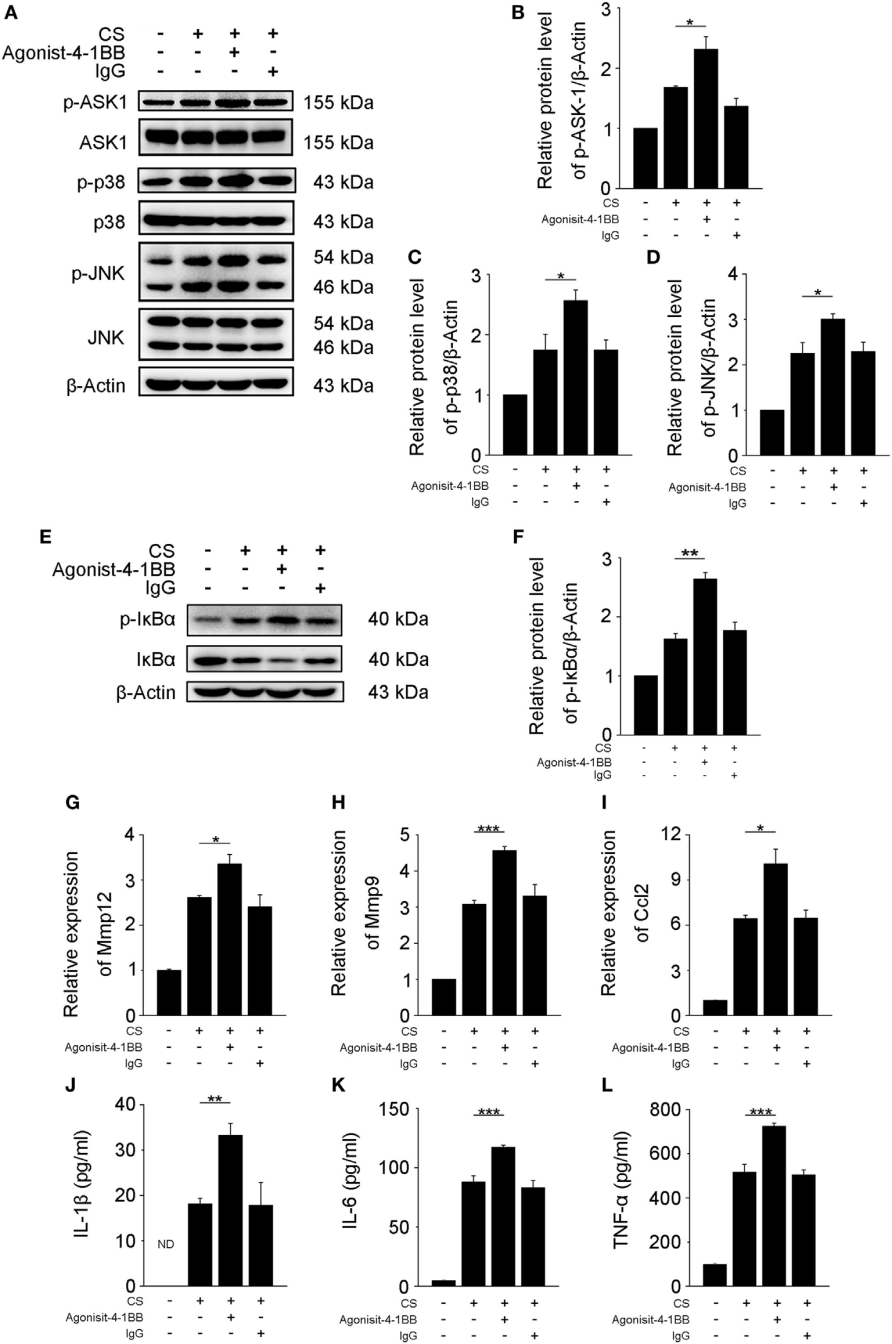
Activation of 4-1BB signaling promotes the secretion of pro-fibrotic mediators by MH-S cells. MH-S cells treated with or without agonist 4-1BB mAb (10 µg/mL) or IgG (10 µg/mL) for 2 h prior to exposure to crystalline silica (50 µg/cm^2^) for 12 h. **(A)** Western blots analysis of ASK-1 and downstream mitogen-activated protein kinase proteins (p38 and JNK/stress activated protein kinase) and their phosphorylated forms. **(B–D)** The levels of phospho-ASK1, phospho-p38, and phospho-JNK were normalized to those of β-actin (*n* = 3). **(E)** Western blots analysis of IκBα and phospho-IκBα. **(F)** The level of phospho-IκBα was normalized to those of β-actin (*n* = 3). **(G–I)** Real-time polymerase chain reaction analysis of MMP12, MMP9, and monocyte chemoattractant protein-1 mRNA expression (*n* = 4). **(J–L)** ELISA analysis was used to quantify the secretion of IL-1β, IL-6 and tumor necrosis factor-α (*n* = 4). The results were representative of three independent experiments. Results were graphed as the mean ± SEM (*p ≤ 0.05; **p ≤ 0.01; ***p ≤ 0.001).

Previous studies support a role for AMs in the pathogenesis of fibrosis (6, 25). However, the mechanisms by which 4-1BB signaling in AMs affects pro-fibrotic responses are not well understood. An agonist 4-1BB mAb was used to examine the role of 4-1BB in the pro-fibrotic response of MH-S cells. The phosphorylation of ASK-1 and downstream mitogen-activated protein kinase (MAPK) proteins [p38 and c-Jun N-terminal kinase (JNK)/stress-activated protein kinase (SAPK)] was markedly elevated in MH-S cells that were stimulated with CS in the presence of agonist 4-1BB mAb (Figures 4A–D). Agonist 4-1BB mAb promoted the phosphorylation of IκBα in MH-S cells upon exposure to CS, while in contrast, the total protein level of IκBα decreased (Figures 4E,F). Thus, in MH-S cells, 4-1BB signaling can be initiated by an agonist 4-1BB mAb. Macrophages secrete MMPs, including MMP9 and MMP12, which participate in the pathogenesis of pulmonary fibrosis (26, 27). However, whether 4-1BB signaling in macrophages could influence the secretion of MMP9 and MMP12 was unknown. As shown in Figures 4G,H, CS combined with agonist 4-1BB mAb stimulated MH-S cells to express significantly more MMP9 and MMP12 than CS alone. These data suggest that activating 4-1BB signaling in macrophage-like cells enhances the expression of MMP9 and MMP12. After engulfing CS, macrophages may release pro-inflammatory and pro-fibrotic cytokines, and chemokines, including IL-1β, IL-6, TNF-α, and MCP-1, which would exaggerate fibrosis (25). It is known that 4-1BB signaling is associated with the expression of pro-inflammatory mediators, including IL-1β, IL-6, TNF-α, and MCP-1 (21, 28, 29). We found that the relative expression of MCP-1 was markedly upregulated in MH-S cells treated with CS and agonist 4-1BB mAb compared to CS alone (Figure 4I). Similarly, MH-S cells secreted more IL-1β, IL-6, and TNF-α after activation of 4-1BB signaling by agonist 4-1BB mAb (Figures 4J–L). Together, these data demonstrate that activating 4-1BB signaling in macrophage-like cells results in the promotion of pro-fibrotic responses after exposure to CS.

### Blocking 4-1BB Signaling Alleviates Pro-Fibrotic Responses of Macrophages

**Figure 5:**
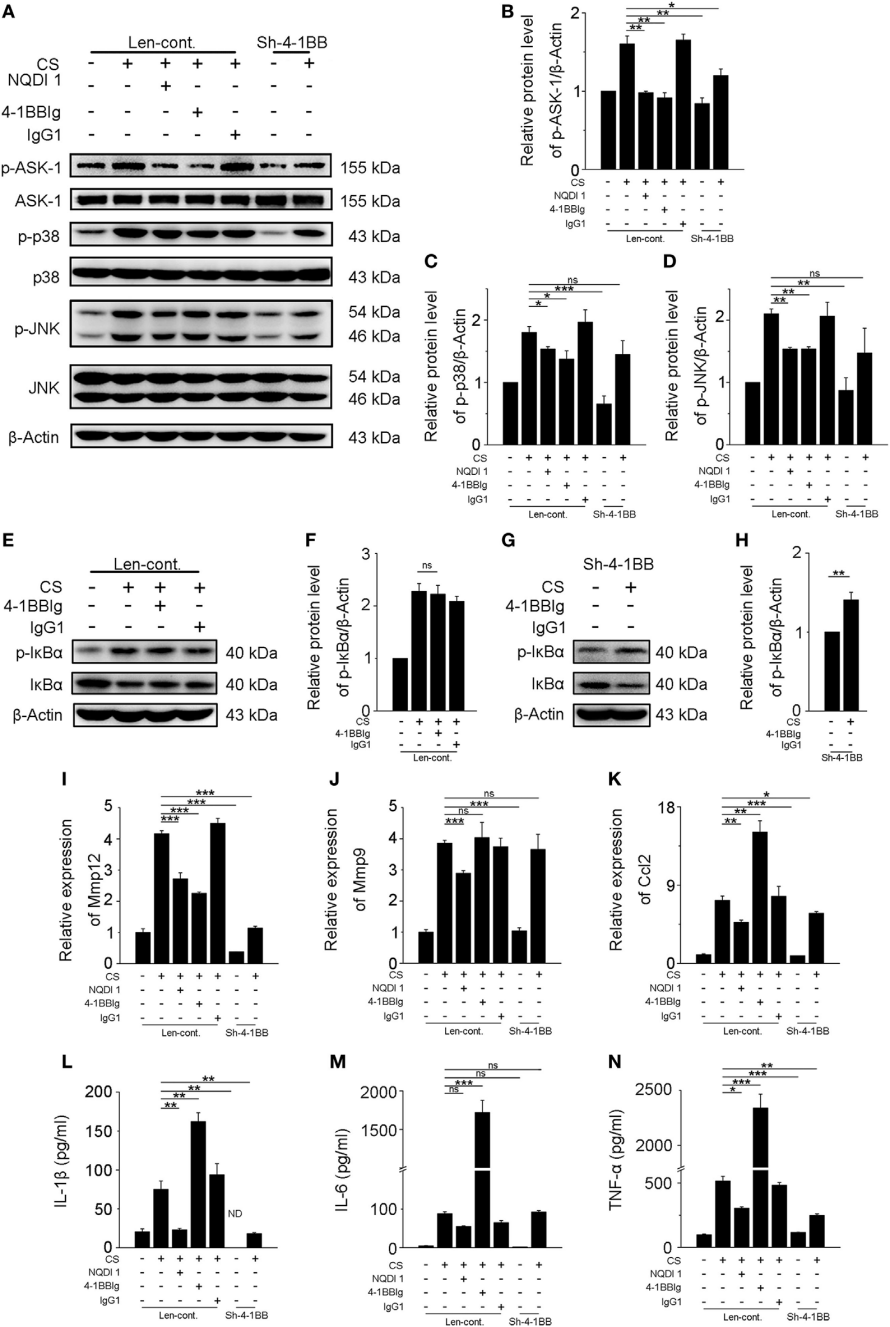
Blockade of 4-1BB signaling attenuates the secretion of pro-fibrotic mediators by MH-S cells. Transfected (Len-cont. and sh-4-1BB) MH-S cells were treated with or without NQDI 1 (10 µM), 4-1BBIg (10 µg/mL), or IgG1 (10 µg/mL) for 2 h, then exposed to crystalline silica (50 µg/cm^2^) for 12 h. **(A)** Western blots analysis of ASK-1 and downstream mitogen-activated protein kinase proteins (p38 and JNK/stress activated protein kinase) and their phosphorylated forms. **(B–D)** Levels of phospho-ASK1, phospho-p38, and phospho-JNK were normalized to those of β-actin (*n* = 3). **(E,G)** Western blots analysis of IκBα and phospho-IκBα. **(F,H)** The level of phospho-IκBα was normalized to those of β-actin (*n* = 3). **(I–K)** The expressions of MMP12, MMP9, and monocyte chemoattractant protein-1 were detected by real-time polymerase chain reaction analysis (*n* = 4). ELISA analysis of cytokines in the culture supernatants. **(L)** IL-1β, **(M)** IL-6, **(N)** tumor necrosis factor-α (*n* = 4). Data were shown as mean ± SEM (*p ≤ 0.05; **p ≤ 0.01; ***p ≤ 0.001; ns, not significant). The data were representative of three independent experiments.

Previous results showed that activating 4-1BB signaling in macrophages promoted pro-fibrotic responses. To determine whether the inhibition of 4-1BB signaling could block CS-induced macrophage pro-fibrotic responses, we used three approaches: treatment with NQDI 1, a specific inhibitor of ASK-1 downstream of 4-1BB (20); 4-1BBIg that can interact with 4-1BBL (as shown in Figures S2B,C in Supplementary Material) to suppress 4-1BB signaling (21); and, as a positive control, MH-S cells in which 4-1BB expression was silenced (Sh-4-1BB, Figures S3B–D in Supplementary Material). The protein levels of ASK-1 and downstream phosphorylated MAPK proteins (p38 and JNK/SAPK) were downregulated in all cases (Figures 5A–D). 4-1BBIg had no effect on the phosphorylation of IκBα in MH-S cells treated with CS (Figures 5E,F). As shown in Figures 5G,H, CS treatment increased the phosphorylation of IκBα in 4-1BB silenced macrophages. These data imply that 4-1BB signaling in MH-S cells can be blocked by treatment with NQDI 1 or 4-1BBIg and 4-1BB silencing. Compared with exposure to CS alone, the expression of MMP12 was significantly attenuated in MH-S cells in which the expression of 4-1BB was silenced or after treatment with NQDI 1 or 4-1BBIg (Figure 5I). In contrast, we found that NQDI 1 markedly inhibited the expression of MMP9, while 4-1BBIg and silencing 4-1BB expression did not (Figure 5J). These results suggest that the various ways of inhibiting 4-1BB signaling may influence macrophage-like cells differently. We further measured the expressions of IL-1β, IL-6, TNF-α, and MCP-1 regulated by 4-1BB signaling in MH-S cells. As illustrated in Figure 5K, the relative expression of MCP-1 was notably alleviated by silencing 4-1BB expression and after NQDI 1 treatment, while it was prominently increased upon treatment with 4-1BBIg. Consistent with this finding, both NQDI 1 treatment and silencing of 4-1BB expression suppressed the CS-induced secretion of pro-inflammatory and pro-fibrotic cytokines, including IL-1β and TNF-α, but not IL-6. In contrast, 4-1BBIg promoted the secretion of IL-1β, IL-6, and TNF-α relative to CS treatment alone (Figures 5L–N). These data imply that blocking 4-1BB signaling in macrophage-like cells may restrain the release of pro-fibrotic mediators after exposure to CS.

### Blockade of 4-1BB Signaling Reduces Pulmonary Fibrosis Responses in CS-Injured Mice

**Figure 6:**
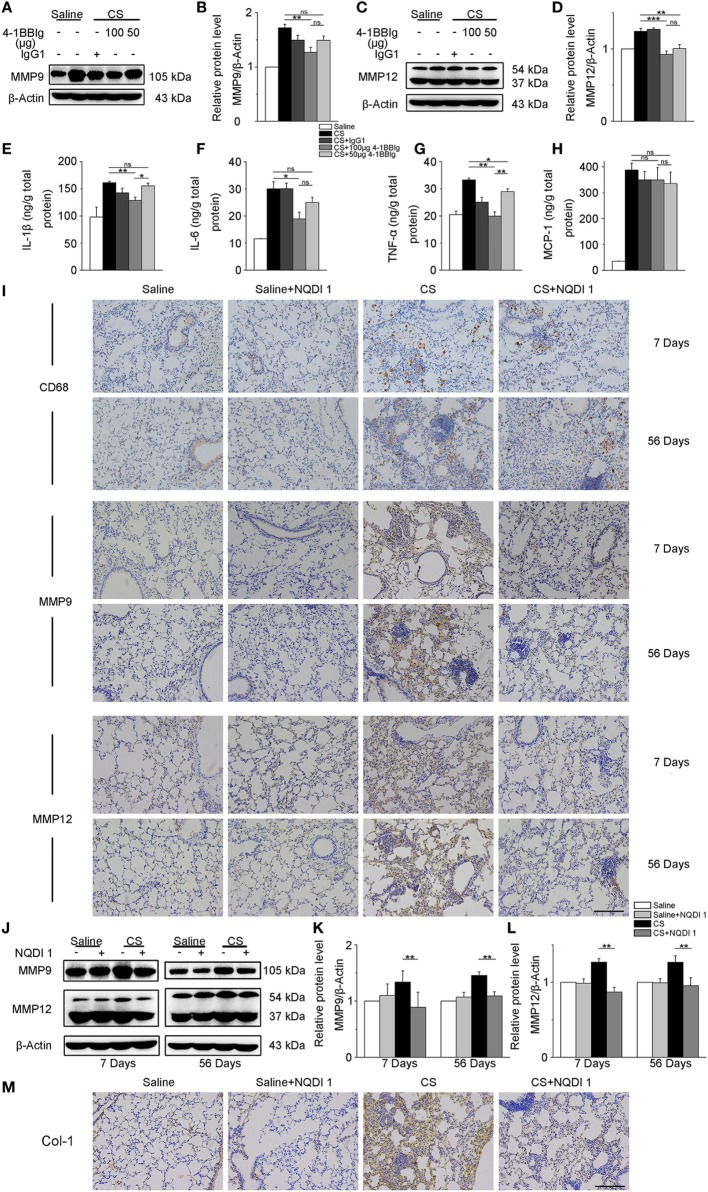
The secretion of pro-fibrotic mediators is reduced in the lungs from crystalline silica (CS)-injured mice, upon inhibition of 4-1BB signaling. C57BL/6 mice were administered a CS suspension or saline, respectively; 4-1BBIg or isotype control (IgG1) were injected intraperitoneally (i.p.; *n* = 3–4). **(A–D)** Quantification of MMP9 and MMP12 protein levels by western blot, which were normalized to those of β-actin in lungs. Shown as bar graph. **(E–H)** ELISA analysis of cytokines in lung tissues. **(E)** IL-1β, **(F)** IL-6, **(G)** tumor necrosis factor-α, **(H)** monocyte chemoattractant protein-1. Experiments were performed three times. C57BL/6 mice were administered a CS suspension or saline, respectively; NQDI 1 or isotype control were injected i.p. (*n* = 10). **(I)** Immunohistochemical staining of paraffin-embedded lung tissue sections at 7 and 56 days showed CD68, MMP9, and MMP12 expression. Nuclei were stained by hematoxylin (blue). **(J–L)** Identification of MMP9 and MMP12 protein levels in mouse lung tissues at 7 and 56 days by western blot. The levels of MMP9 and MMP12 were normalized to those of β-actin. **(M)** Representative images for the immunohistochemical staining of collagen I in paraffin-embedded lung tissue sections 56 days after CS instillation. Nuclei were stained by hematoxylin (blue). **(I,M)** Scale bar, 50 µm. Experiments were performed three times. Data are shown as mean ± SEM (*p ≤ 0.05; **p ≤ 0.01; ***p ≤ 0.001; ns, not significant).

Given our previous *in vitro* results, we wondered whether these same 4-1BB blocking treatments could attenuate pro-fibrotic responses in mice. To examine the effect of 4-BBIg in CS-injured mice, we treated mice with different doses of 4-1BBIg, and then examined 4-1BB downstream signaling and the expression of pro-fibrotic mediators. The phosphorylation of p38 and JNK decreased, upon 4-1BBIg treatment (Figure S5 in Supplementary Material). Western blot analysis indicated that CS-injured mice treated with 100 µg 4-1BBIg exhibited a dramatic reduction in protein levels of MMP9 and MMP12 (Figures 6A–D). CS-injured mice treated with 100 µg 4-1BBIg had markedly lower levels of IL-1β, IL-6, and TNF-α in their lungs (Figures 6E–G). As shown in Figure 6H, the level of MCP-1 did not differ among the groups of mice. These results suggest that 4-1BBIg can block 4-1BB signaling and influence the reduction of pro-fibrotic mediators *in vivo*.

Next, we treated mice with NQDI 1 to validate its inhibitory effect *in vivo*. Immunohistochemistry analysis illustrated that NQDI 1 treatment could reduce CD68+ macrophages accumulation, MMP9 and MMP12 expressions in the lungs at 7 and 56 days (Figure 6I). In accordance with these results, MMP9 and MMP12 protein levels were noticeably downregulated in NQDI 1-treated CS-injured mice at different time points (Figures 6J–L). These data indicate that NQDI 1 may reduce pro-fibrotic mediators *in vivo*. The deposition of type 1 collagen was markedly reduced in the lungs of mice treated with NQDI 1 (Figure 6M). Our results suggest that blocking ASK-1/p38/JNK pathway, downstream of 4-1BB signaling, with NQDI 1 can reduce pro-fibrotic mediators *in vivo*.

## Discussion

**Figure 7:**
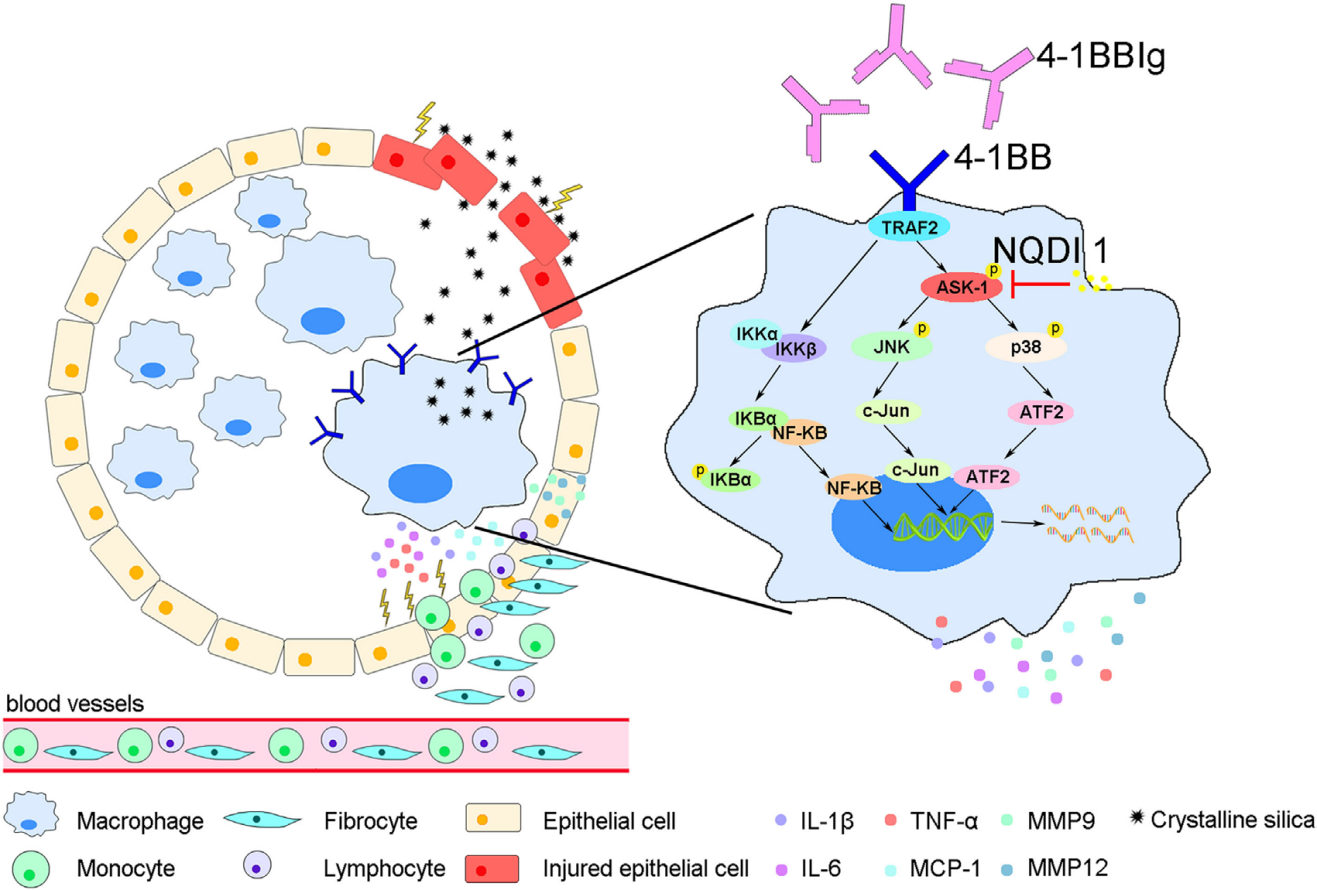
A model for alveolar macrophages (AMs) expressing 4-1BB in the regulation of pulmonary fibrosis through secreting pro-fibrotic mediators. The expression of 4-1BB increases in AMs in response to crystalline silica, which leads to elevated secretion of pro-inflammatory and pro-fibrotic cytokines, chemokines, and MMPs. These pro-fibrotic mediators promote pulmonary alveoli injury, the accumulation of monocytes, lymphocytes, and fibrocytes, and collagen deposition, resulting in pulmonary fibrosis.

In this study, we showed that 4-1BB levels increase on the surface of AMs in a mouse model of experimental silicosis. We then verified that 4-1BB signaling in macrophage-like cells affected the secretion of pro-inflammatory and pro-fibrotic cytokines, chemokines, and MMPs after exposure to CS. Furthermore, we showed that the pharmacological blockade of 4-1BB signaling alleviated CS-induced pulmonary fibrosis responses *in vivo*. Therefore, we speculate that AMs expressing 4-1BB secrete pro-fibrotic mediators, which orchestrate a pro-fibrotic microenvironment to promote pulmonary fibrosis (Figure 7).

Our previous study showed that the expression of 4-1BB was dramatically upregulated in CS-injured mice relative to control mice after 7 and 56 days (17). It remained to be determined what cells express 4-1BB in CS-injured lungs. Macrophages, are recognized as antigen-presenting cells that express 4-1BBL to activate T cells, rather than expressing 4-1BB (23). However, recently, Jung et al. reported that 4-1BB could be expressed on macrophages and promoted the destabilization of atherosclerotic plaques (14). We verified the expression of 4-1BB in pulmonary macrophages (including AMs and IMs), which play a critical role at all stages of pulmonary fibrosis (25). After CS inhalation, the number of AMs and IMs was elevated in mice lungs, but only the percentage of AMs within CD45+ cells increased. 4-1BB was more highly expressed in AMs from CS-injured mice, while IMs were not significantly affected upon CS exposure (Figure 1). CD4+ T cells expressing 4-1BB have been studied in numerous disease models (12). We further tested the expression of 4-1BB in CD4+ T cells (including effector and naïve T cells), which are involved in CS-induced inflammation and pulmonary fibrosis (30). The number of effector T cells was upregulated in lungs from CS-injured mice compared with controls. However, the percentage of effector T cells expressing 4-1BB was no different between CS-injured mice and controls (Figure 2). We reasoned that AMs may be the predominant cell-type to increase 4-1BB expression in CS-injured mice. Therefore, we used MH-S cells (an *in vitro* model of AMs) as our major study subject for the *in vitro* experiments.

4-1BB can interact with 4-1BBL, which results in bidirectional signal. After aggregation, TRAF2 is recruited, leading to the activation of ASK-1/p38/JNK pathway (31). In our previous study, we found that the phosphorylation of ASK-1 was reduced *in vitro* and *in vivo*, dependent on 4-1BBIg concentration (17). Our data indicated that 4-1BB might play an important role in triggering ASK-1 activation. NQDI 1, a selective inhibitor of ASK-1, did not affect the interaction between 4-1BB and 4-1BBL. Thus, we used NQDI 1 to block 4-1BB signaling *in vitro* and *in vivo*. 4-1BB could also activate NF-κB pathway after interacting with 4-1BBL (32). In our present study, we found that agonist 4-1BB mAb promoted the phosphorylation of IκBα (Figure 4), which was consistent with previous studies (32). Nevertheless, 4-1BBIg had no effect on the phosphorylation of IκBα (Figure 5). Previous research suggests that 4-1BB fusion protein (4-1BBIg) interacts with 4-1BBL and induces nuclear translocation of NF-κB in human monocytes (31). Besides, 4-1BBIg could inhibit 4-1BB signaling, which might lead to suppressing NF-kB activation. Therefore, we reasoned that 4-1BBIg treatment could not significantly influence NF-kB activation.

Alveolar macrophages are the first line of immune defense against CS (8). These cells secrete pro-inflammatory and pro-fibrotic cytokines and chemokines, including IL-1β, IL-6, TNF-α, and MCP-1, which influence the pathogenesis of pulmonary fibrosis (33–36). These cytokines can cause epithelial barrier loss, induce fibroblasts activation, and promote the recruitment of monocytes, lymphocytes, and fibroblasts (37–40). In the present study, we found that MH-S cells released higher levels of IL-1β, IL-6, TNF-α, and MCP-1 upon activation of 4-1BB signaling (Figure 4). This was in agreement with previous studies in adipocytes and myocardial cells showing that activating 4-1BB signaling enhanced the secretion of these inflammatory cytokines (21, 28). We then used two inhibitors of 4-1BB signaling (4-1BBIg, upstream blocking; NQDI 1, downstream blocking) to further identify the effect of 4-1BB signaling on cytokine secretion. NQDI 1 alleviated cytokine secretion in macrophage-like cells. However, to our surprise, 4-1BBIg treatment promoted the secretion of IL-1β, IL-6, TNF-α, and MCP-1 in MH-S cells (Figure 5), but not in CS-injured mice, in which we found decreased levels of these cytokines. According to our results (Figure 1), we found that CS stimulation could upregulate the expressions of both 4-1BB and 4-1BBL molecules on AMs, which can interact with each other leading to activation of a bidirectional signal. We speculate that 4-1BBIg may sustain macrophage proliferation/survival, differentiation, and secretion of pro-inflammatory cytokines by interacting with 4-1BBL, also supported by other researches (23, 41, 42). In addition, we found that CS treatment promoted 4-1BBL expression in MH-S cells, inhibited by 4-1BBIg treatment (Figure 2). It might be possible that 4-1BBIg downregulates 4-1BBL expression (since 4-1BBIg can bind to 4-1BBL) resulting in the increased secretion of pro-inflammatory cytokines. However, in our previous study, we found that 4-1BBIg upregulated the expression of 4-1BBL in cocultures of lymphocytes and lung single cells, whereas it did not affect the expression of 4-1BBL in lungs from CS-injured mice *in vivo* (17). We then suggest that *in vitro* 4-1BBIg treatment might have different effects from *in vivo* treatment. Perhaps, the concentration of 4-1BBIg used *in vivo* was not high enough to stimulate 4-1BBL signaling, while it blocked 4-1BB signaling.

Alveolar macrophages are important producers of MMPs, including MMP9 and MMP12, which are vital players in pulmonary fibrosis (6, 43). Previous studies have shown that 4-1BB signaling regulates the secretion of MMP9 and MMP12 (14, 44). In this study, we showed that activating 4-1BB signaling in macrophage-like cells increased the expressions of MMP9 and MMP12 (Figure 4), opposite to 4-1BB-blockade effects (Figure 5). MMP9 and MMP12 play a central role in inflammatory responses, induced in response to damage-associated molecules released by injured lung tissues, and subsequently affect the development of fibrosis (45, 46). At early inflammatory stage, MMP9 and MMP12 may degrade the basement membrane and increase the activity of inflammatory cytokines and chemokines (47), which then upregulate inflammatory responses. Previous studies have shown that the severity of inflammatory responses markedly decreased in MMP9^−/−^ asthma mouse models (26), and IL-13-induced inflammation reduced upon MMP12 knockdown in mice (48). In the present study, CS-injured mice treated with NQDI 1 or 4-1BBIg had lower levels of MMP9 and MMP12 after 7-days exposure to CS (Figure 6). Along with our previous study (17), we speculate that the reduction of MMP9 and MMP12 consequent to inhibition of 4-1BB signaling reduces tissue injury and alleviates the recruitment of immune cells, leading to downregulated CS-induced inflammatory responses. Previously, TGF-β was shown to increase in CS-injured mice (22), promoting the differentiation and proliferation of myofibroblasts and aggravating fibrosis (49). At tissue repair stage, MMP9 and MMP12 can enhance the secretion and maturity of TGF-β *via* cleaving the inactive complex, consisting of TGF-β, TGF-β latency-associated protein, and latent TGF-β-binding protein (50); such molecules can also add to the deposition of collagen type I, a part of ECM protein (51). Our findings showed that the expression of MMP9 and MMP12 and the deposition of collagen type I were reduced in mice treated with NQDI 1 after 56-days exposure to CS. We speculate that inhibition of MMP9 and MMP12 induced by blocking 4-1BB signaling attenuates CS-induced pulmonary fibrosis *via* lessening the maturity of TGF-β and the deposition of collagen type I. Future studies are needed to address this hypothesis.

In summary, the findings from this study support an essential role for 4-1BB signaling modulating pro-fibrotic responses when responding to stimulation by CS particles *in vitro* and *in vivo*. 4-1BB signaling might mediate pulmonary fibrosis by promoting three different pathways: (i) secretion of pro-inflammatory and pro-fibrotic cytokines that affect pulmonary alveoli injury, and the proliferation and differentiation of fibroblasts; (ii) release of chemokines that recruit immune cells and fibrocytes to injured tissues; and (iii) upregulation of the expression of MMPs that support inflammation and collagen deposition. Therefore, we speculate that blocking 4-1BB signaling could be considered as a potential therapeutic target to treat CS-induced pulmonary fibrosis.

## Ethics Statement

This study was carried out in accordance with the recommendations of the National Institute of Health Guide for the Care and Use of Laboratory Animals and Animal Care and Use Committee at the China Medical University. The protocol was approved by the Animal Care and Use Committee at the China Medical University.

## Author Contributions

YL, CL, and JC were responsible for the conception and design of the study. YL, SD, XC, and XZ performed experiments. YL and CL analyzed results and interpreted data. JC supervised the study. YL and CL drafted the manuscript. FL, YC, and JC helped to revise the manuscript. All authors read and approved the final manuscript.

## Conflict of Interest Statement

The authors declare that the research was conducted in the absence of any commercial or financial relationships that could be construed as a potential conflict of interest.

## Supplementary Material

Figure S1 Immunophenotyping gating strategy for pulmonary macrophages and CD4+ T cells. Gating strategies for flow cytometric analyses in a subject were shown as an example. Lung single-cells were initially gated on singlet cells by forward-scatter (FSC)-area (FSC-A) and FSC-height (FSC-H), then by FSC-A and sidescatter (SSC)-area (SSC-A). AMs were defined as CD45+F4/80+CD11c+; IMs as CD45+F4/80+CD11c−; effector T cells as CD4+CD44+CD62L−, and naïve T cells as CD4+CD44−CD62L+. Then, cells were examined for the expression of 4-1BB or 4-1BBL. All isotype-matched antibodies were used as negative controls.

Figure S2 4-1BBL expression on MH-S cells. **(A)** Gating strategies for flow cytometric analyses in a subject were shown as an example. MH-S cells were gated on singlet cells by FSC-A and FSC-H, then by FSC-A and SSC-A. Isotype-matched antibody was used as a negative control. MH-S cells were treated with or without 4-1BBIg (10 µg/mL) or IgG1 (10 µg/mL) for 2 h, then exposed to crystalline silica (50 µg/cm^2^) for 12 h. **(B,C)** The percentage of MH-S cells expressing 4-1BBL (*n* = 4). Data were shown as mean ± SEM (****p* ≤ 0.001). The data were representative of three independent experiments.

Figure S3 Effect of lentiviral 4-1BB shRNA transfection on 4-1BB expression in MH-S cells. **(A)** MH-S cells were transfected with Lentiviral-4-1BB-shRNA at different MOIs (10, 30 and 50, respectively) over 48 h. MH-S cells that transfected with or without vectors with shRNAs against 4-1BB (sh-4-1BB) were treated with crystalline silica (CS) (50 µg/cm^2^) for 12 h. **(B,C)** The percentage of MH-S cells expressing 4-1BB (*n* = 4). **(D)** MH-S cells, transfected with or without lentiviral control vectors or lentiviral 4-1BB shRNA, were treated with or without CS (50 µg/cm^2^) for 12 h. Identification of the protein level of 4-1BB (*n* = 3). Results showed as mean ± SEM (***p* ≤ 0.01). Data were representative of three independent experiments.

Figure S4 Identification of the NQDI 1 dose. MH-S cells were pretreated with different doses of NQDI-1 (1, 3, 5 and 10 µM) for 2 h, and then were treated with or without crystalline silica (50 µg/cm^2^) for 12 h. Western blots of ASK-1 downstream mitogen-activated protein kinase proteins (p38 and JNK/stress activated protein kinase) in MH-S cells (*n* = 3). Experiments were performed three times.

Figure S5 4-1BBIg blocked the 4-1BB pathway in crystalline silica (CS) injured mice. C57BL/6 mice were administered a CS suspension or saline, respectively; 4-1BBIg (50 or 100 µg) or isotype control were injected i.p. Western blots of ASK1 downstream mitogen-activated protein kinase proteins (p38 and JNK/stress activated protein kinase) in the lung tissues (*n* = 3). Data were representative of three independent experiments.
