# Supplementary figures and images for: 4-1BB Signaling Promotes Alveolar Macrophages-Mediated Pro-Fibrotic Responses and Crystalline Silica-Induced Pulmonary Fibrosis in Mice
Source: Front Immunol. 2018 Sep 10;9:1848. doi: 10.3389/fimmu.2018.01848 (PMC6139304; doi:10.3389/fimmu.2018.01848)

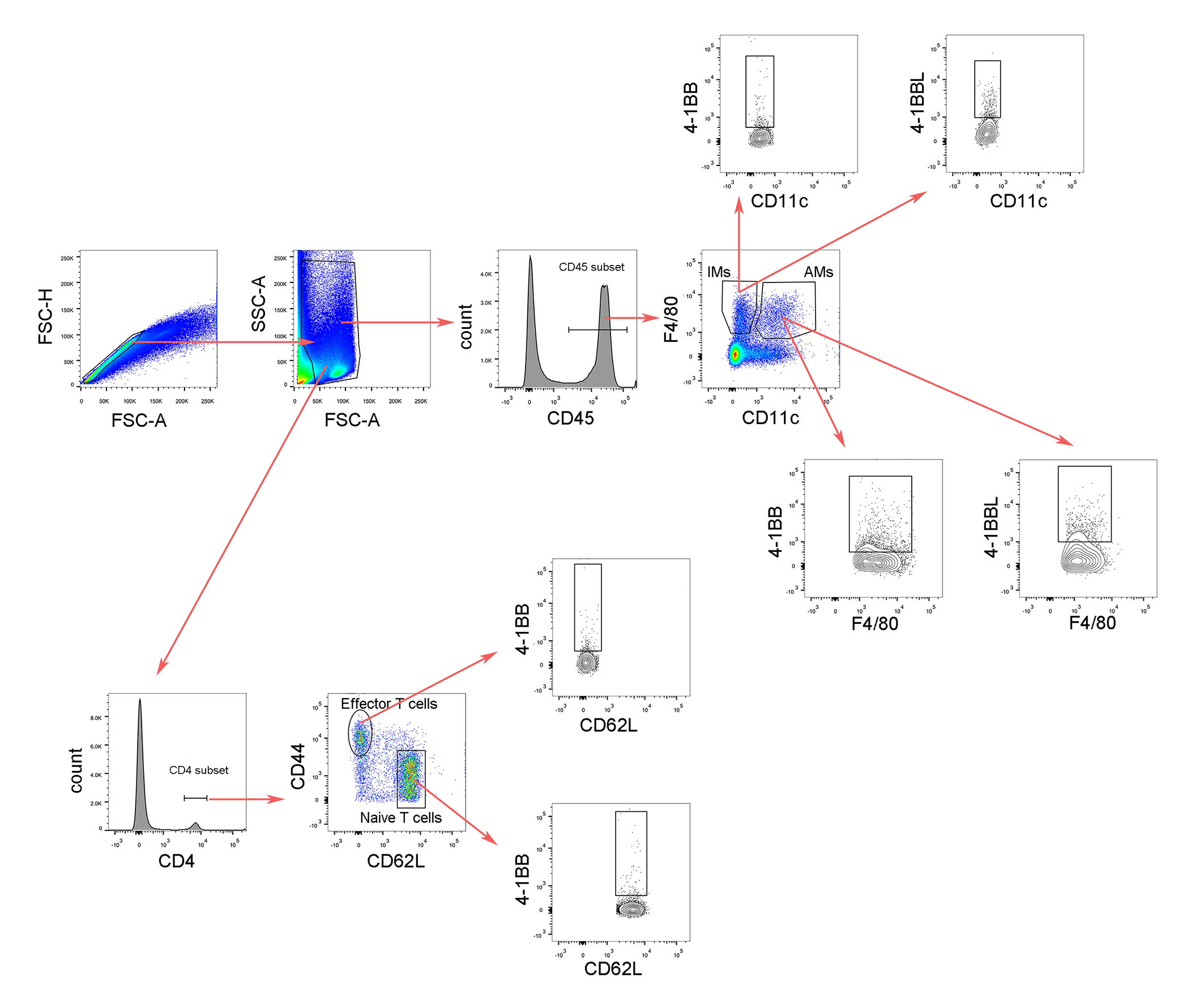

Supplement: Figure S1 — Immunophenotyping gating strategy for pulmonary macrophages and CD4+ T cells. Gating strategies for flow cytometric analyses in a subject were shown as an example. Lung single-cells were initially gated on singlet cells by forward-scatter (FSC)-area (FSC-A) and FSC-height (FSC-H), then by FSC-A and sidescatter (SSC)-area (SSC-A). AMs were defined as CD45+F4/80+CD11c+; IMs as CD45+F4/80+CD11c−; effector T cells as CD4+CD44+CD62L−, and naïve T cells as CD4+CD44−CD62L+. Then, cells were examined for the expression of 4-1BB or 4-1BBL. All isotype-matched antibodies were used as negative controls. [file image_1.tif]

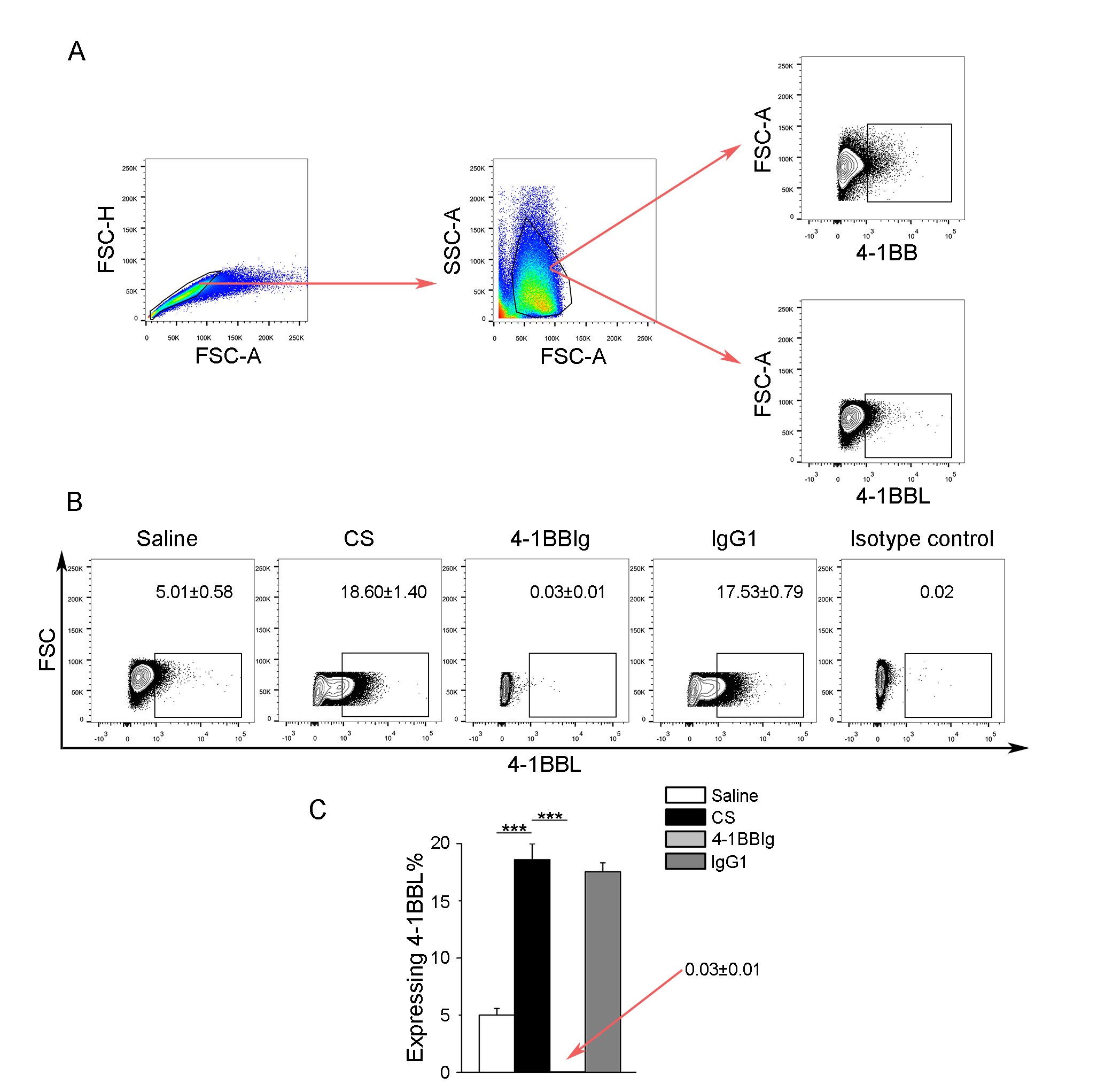

Supplement: Figure S2 — 4-1BBL expression on MH-S cells. (A) Gating strategies for flow cytometric analyses in a subject were shown as an example. MH-S cells were gated on singlet cells by FSC-A and FSC-H, then by FSC-A and SSC-A. Isotype-matched antibody was used as a negative control. MH-S cells were treated with or without 4-1BBIg (10 µg/mL) or IgG1 (10 µg/mL) for 2 h, then exposed to crystalline silica (50 µg/cm2) for 12 h. (B,C) The percentage of MH-S cells expressing 4-1BBL (n = 4). Data were shown as mean ± SEM (***p ≤ 0.001). The data were representative of three independent experiments. [file image_2.tif]

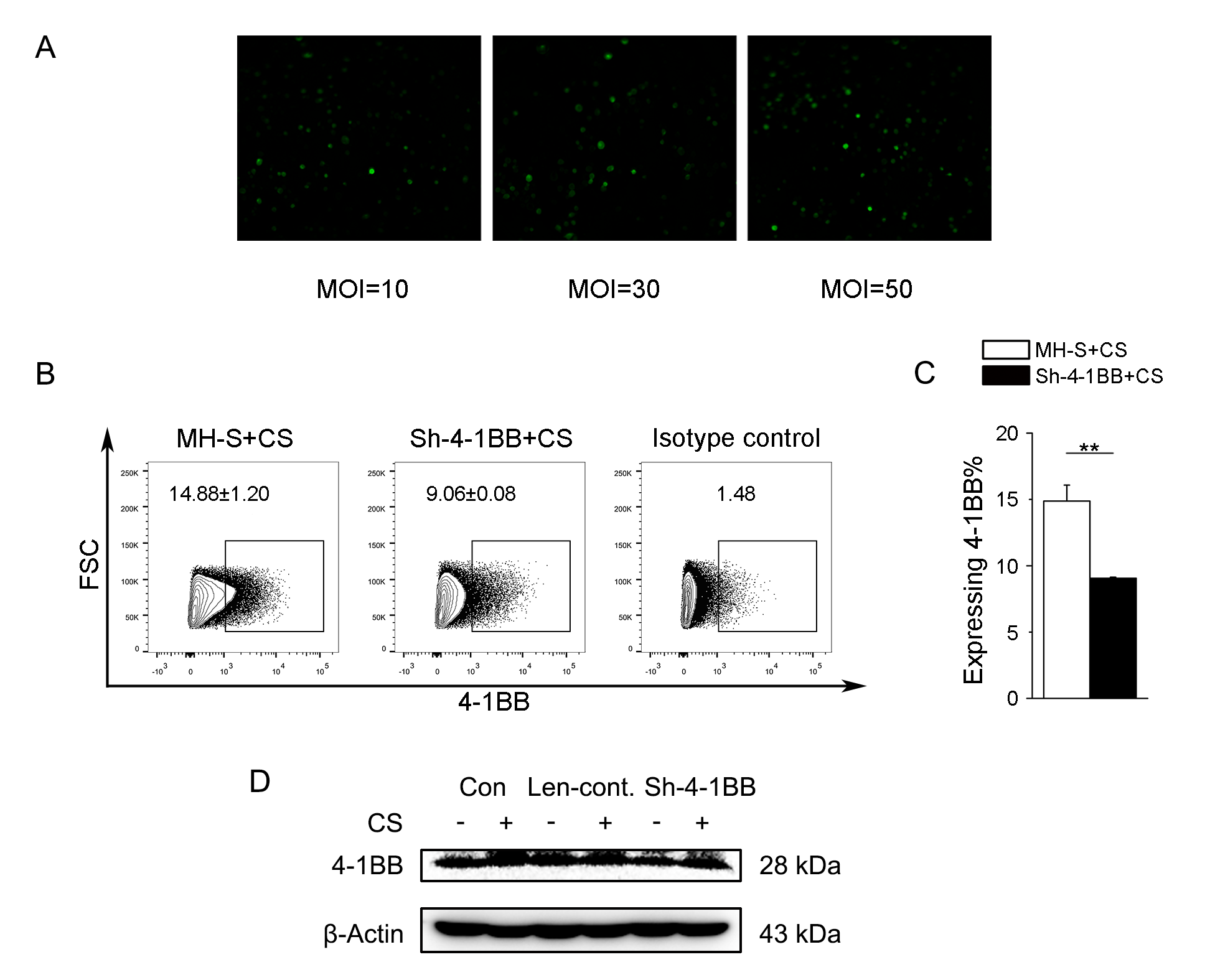

Supplement: Figure S3 — Effect of lentiviral 4-1BB shRNA transfection on 4-1BB expression in MH-S cells. (A) MH-S cells were transfected with Lentiviral-4-1BB-shRNA at different MOIs (10, 30 and 50, respectively) over 48 h. MH-S cells that transfected with or without vectors with shRNAs against 4-1BB (sh-4-1BB) were treated with crystalline silica (CS) (50 µg/cm2) for 12 h. (B,C) The percentage of MH-S cells expressing 4-1BB (n = 4). (D) MH-S cells, transfected with or without lentiviral control vectors or lentiviral 4-1BB shRNA, were treated with or without CS (50 µg/cm2) for 12 h. Identification of the protein level of 4-1BB (n = 3). Results showed as mean ± SEM (**p ≤ 0.01). Data were representative of three independent experiments. [file image_3.tif]

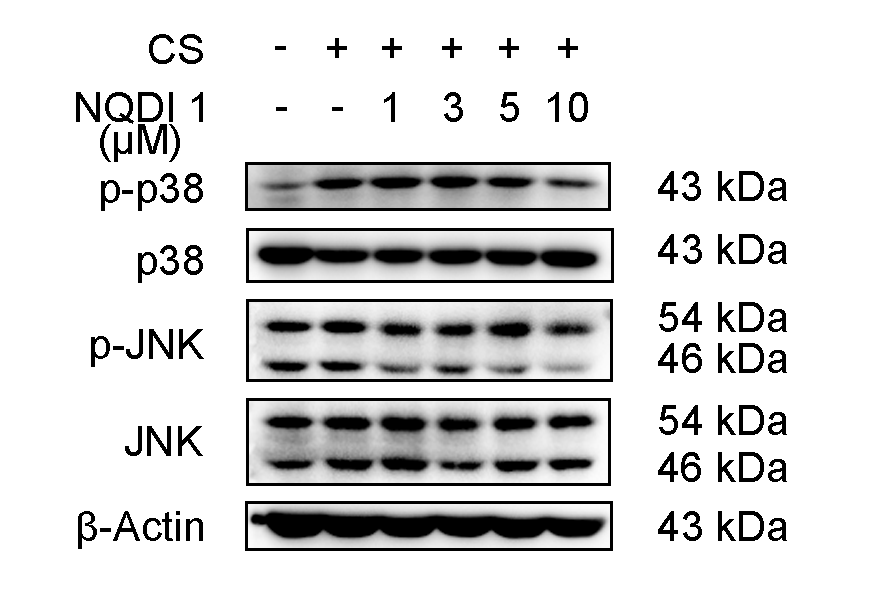

Supplement: Figure S4 — Identification of the NQDI 1 dose. MH-S cells were pretreated with different doses of NQDI-1 (1, 3, 5 and 10 µM) for 2 h, and then were treated with or without crystalline silica (50 µg/cm2) for 12 h. Western blots of ASK-1 downstream mitogen-activated protein kinase proteins (p38 and JNK/stress activated protein kinase) in MH-S cells (n = 3). Experiments were performed three times. [file image_4.tif]

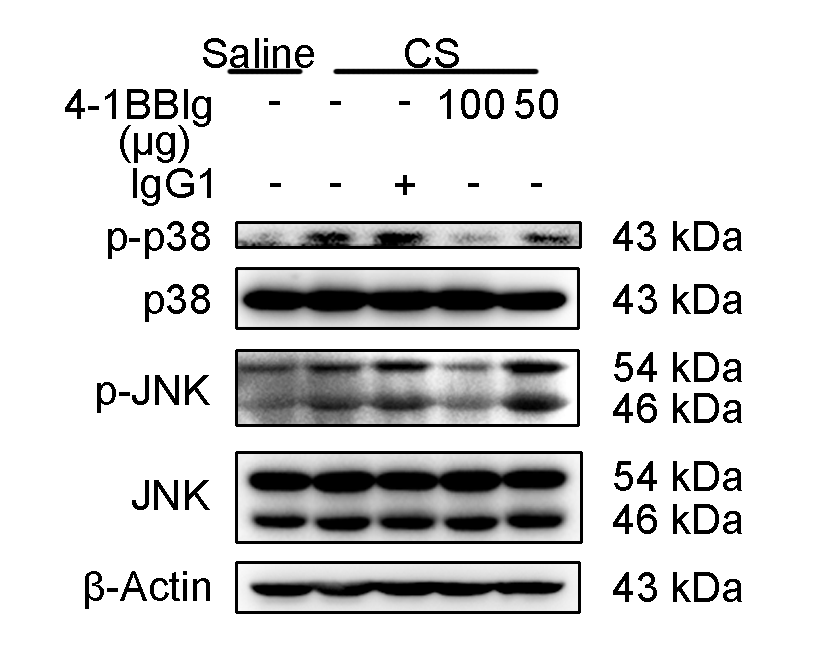

Supplement: Figure S5 — 4-1BBIg blocked the 4-1BB pathway in crystalline silica (CS) injured mice. C57BL/6 mice were administered a CS suspension or saline, respectively; 4-1BBIg (50 or 100 µg) or isotype control were injected i.p. Western blots of ASK1 downstream mitogen-activated protein kinase proteins (p38 and JNK/stress activated protein kinase) in the lung tissues (n = 3). Data were representative of three independent experiments. [file image_5.tif]
