# Supplementary material for: 4-1BB Signaling Promotes Alveolar Macrophages-Mediated Pro-Fibrotic Responses and Crystalline Silica-Induced Pulmonary Fibrosis in Mice
Source: Front Immunol. 2018 Sep 10;9:1848. doi: 10.3389/fimmu.2018.01848 (PMC6139304; doi:10.3389/fimmu.2018.01848)
Supplement: Supplementary file 6 [file table_1.docx]

Table S1. Sources of antibodies used in the study

| **methods** | **antibodies** | **Clone** | **Catalog** **number** | **Vender** |
| --- | --- | --- | --- | --- |
| **flow cytometry** | Rat anti-mouse CD16/CD32 | 2.4G2 | 553141 | BD Biosciences |
|  | CD45-PerCP-Cy5.5 | 30-F11 | 550994 | BD Biosciences |
|  | CD11c-FITC | HL3 | 557400 | BD Biosciences |
|  | CD4-PerCP-Cy5.5 | RM4-5 | 550954 | BD Biosciences |
|  | CD44-FITC | IM7 | 561859 | BD Biosciences |
|  | CD62L-APC | MEL-14 | 561919 | BD Biosciences |
|  | F4/80-APC | REA126 | 130­102­379 | Miltenyi Biotech |
|  | CD137-PE | 17B5 | 12-1371 | eBioscience™ |
|  | CD137L-PE | TKS-1 | 12-5901 | eBioscience™ |
|  | PE-Rat IgG2a isotype control | eBR2a | 12-4321 | eBioscience™ |
|  | PE-Syrian Hamster IgG isotype control | from normal Golden Syrian hamster serum | 12-4914 | eBioscience™ |
| **western blot** | CD137 | RM0068-4G33 | ab86576 | Abcam |
|  | ASK1 | EP553Y | ab45178 | Abcam |
|  | MMP9 | Polyclonal | ab38898 | Abcam |
|  | MMP12 | EP1261Y | ab52897 | Abcam |
|  | phospho-ASK1 | Polyclonal | 3765S | Cell Signaling Technology |
|  | p38 | D13E1 | 8690S | Cell Signaling Technology |
|  | phospho-p38 | D3F9 | 4511S | Cell Signaling Technology |
|  | JNK | Polyclonal | 9252S | Cell Signaling Technology |
|  | phospho-JNK | 81E11 | 4668S | Cell Signaling Technology |
|  | IκBα | 44D4 | 4812S | Cell Signaling Technology |
| **methods** | **antibodies** | **Clone** | **Catalog** **number** | **Vender** |
| **western blot** | phospho-IκBα | 14D4 | 2859S | Cell Signaling Technology |
|  | β-actin | D6A8 | 8457S | Cell Signaling Technology |
| **immunohistochemistry** | MMP9 | Polyclonal | GB11132 | Servicebio |
|  | CD68 | FA-11 | ab53444 | Abcam |
|  | MMP12 | Polyclonal | ab137444 | Abcam |
|  | collagen Ⅰ | EPR7785 | ab138492 | Abcam |
|  | horseradish peroxidase (HRP) | G-10 | sc-390944 | Santa Cruz Biotechnology |
